# Disease-associated genetic variants in the regulatory regions of human genes: mechanisms of action on transcription and genomic resources for dissecting these mechanisms

**DOI:** 10.18699/VJ21.003

**Published:** 2021-02

**Authors:** E.V. Ignatieva, E.A. Matrosova

**Affiliations:** Institute of Cytology and Genetics of Siberian Branch of the Russian Academy of Sciences, Novosibirsk, Russia Novosibirsk State University, Novosibirsk, Russia; Institute of Cytology and Genetics of Siberian Branch of the Russian Academy of Sciences, Novosibirsk, Russia Novosibirsk State University, Novosibirsk, Russia

**Keywords:** transcription regulation, genetic variability, pathogenic genetic variants, transcription regulatory regions, transcription factor binding sites (TFBSs), genomic databases, регуляция транскрипции, геномная изменчивость, патогенные геномные варианты, районы, регулирующие транскрипцию, сайты связывания транскрипционных факторов, геномные базы данных

## Abstract

Whole genome and whole exome sequencing technologies play a very important role in the studies of the genetic aspects of the pathogenesis of various diseases. The ample use of genome-wide and exome-wide association study methodology (GWAS and EWAS) made it possible to identify a large number of genetic variants associated with diseases. This information is accumulated in the databases like GWAS central, GWAS catalog, OMIM, ClinVar, etc. Most of the variants identified by the GWAS technique are located in the noncoding regions of the human genome. According to the ENCODE project, the fraction of regions in the human genome potentially involved in transcriptional control is many times greater than the fraction of coding regions. Thus, genetic variation in noncoding regions of the genome can increase the susceptibility to diseases by disrupting various regulatory elements (promoters, enhancers, silencers, insulator regions, etc.). However, identification of the mechanisms of influence of pathogenic genetic variants on the diseases risk is difficult due to a wide variety of regulatory elements. The present review focuses on the molecular genetic mechanisms by which pathogenic genetic variants affect gene expression. At the same time, attention is concentrated on the transcriptional level of regulation as an initial step in the expression of any gene. A triggering event mediating the effect of a pathogenic genetic variant on the level of gene expression can be, for example, a change in the functional activity of transcription factor binding sites (TFBSs) or DNA methylation change, which, in turn, affects the functional activity of promoters or enhancers. Dissecting the regulatory roles of polymorphic loci have been impossible without close integration of modern experimental approaches with computer analysis of a growing wealth of genetic and biological data obtained using omics technologies. The review provides a brief description of a number of the most well-known public genomic information resources containing data obtained using omics technologies, including (1) resources that accumulate data on the chromatin states and the regions of transcription factor binding derived from ChIP-seq experiments; (2) resources containing data on genomic loci, for which allele-specific transcription factor binding was revealed based on ChIP-seq technology; (3) resources containing in silico predicted data on the potential impact of genetic variants on the transcription factor binding sites

## Abstract (ru)

Полногеномные и полноэкзомные технологии секвенирования играют важную роль в исследованиях генетических аспектов патогенеза различных заболеваний. Широкое применение методов полногеномного и полноэкзомного анализа ассоциаций позволяет идентифицировать множество вариантов геномной изменчивости (ГИ), ассоциированных с заболеваниями. Эта информация накапливается в базах данных GWAS central, GWAS catalog, OMIM, ClinVar и др. Большинство вариантов, идентифицированных методикой полногеномного анализа ассоциаций, располагается в некодирующих областях генома человека. По данным проекта ENCODE, доля участков в геноме человека, потенциально задействованных в регуляции транскрипции, во много раз превышает долю кодирующих областей. Таким образом, геномная изменчивость в некодирующих областях генома может повышать предрасположенность к заболеваниям, нарушая функционирование различных регуляторных элементов (промоторов, энхансеров, участков, определяющих 3D структуру хроматина и т. д.). Однако идентификация механизмов влияния патогенных вариантов ГИ на риск развития заболеваний затруднена ввиду большого разнообразия регуляторных элементов. В обзоре рассмотрены молекулярно-генетические механизмы влияния патогенных вариантов ГИ на экспрессию генов. При этом внимание сосредоточено на транскрипционном уровне регуляции как ключевой стадии, запускающей последовательность этапов экспрессии любого гена. Пусковым событием, опосредующим влияние патогенного варианта ГИ на уровень экспрессии гена, может быть, например, изменение функциональной активности сайтов связывания транскрипционных факторов или уровня метилирования ДНК, что, в свою очередь, отражается на функциональной активности промоторов или энхансеров. Выявление регуляторных эффектов полиморфных локусов невозможно без тесной интеграции современных экспериментальных подходов с компьютерным анализом больших массивов генетических данных, получаемых на основе омиксных технологий. В обзоре кратко описаны наиболее известные открытые полногеномные информационные ресурсы, содержащие данные, полученные на основе омиксных технологий, в том числе: ресурсы, накапливающие сведения о состоянии хроматина и участках его связывания с транскрипционными факторами, выявленными с помощью технологии ChIP-seq; ресурсы по геномным локусам, для которых на основе данных ChIP-seq выявлено аллель-специфичное связывание с транскрипционными факторами; а также ресурсы, содержащие предсказанные in silico данные о потенциальном влиянии геномной изменчивости на сайты связывания транскрипционных факторов.

## Introduction

At present, largely due to the widespread use of the technology of genome-wide and exome-wide association study (GWAS and EWAS), a large number of polymorphisms associated with diseases have been identified. For example, GWAS central (https://www.gwascentral.org/) contains information on more than 70 million associations between ~3.2 million SNPs and 1451 diseases or phenotypic characteristics (Beck et al., 2020). Experimental datasets of comparable volume have been accumulated in a number of other databases on genotype-phenotype associations (GWAS catalog, OMIM, ClinVar, HGMD, PheGenI, EGA, GAD, dbGaP).

Currently, a large amount of experimental data has been obtained about the disease-associated genetic variants(GVs), but the molecular mechanisms underlying these associations are extremely poorly understood. This is due to the fact that only a relatively small proportion of pathogenic GVs is located in the coding regions of the human genome, changes in the nucleotide sequence of which disrupt the structure and function of proteins. A huge mass of polymorphic loci associated with diseases is located in non-coding regions of the genome (introns, 5′- and 3′-f lanking regions of genes, intergenic regions). For example, according to GWAS data, ~90 % of the total number of variants associated with diseases are located in noncoding regions of the human genome (Maurano et al., 2012; Farh et al., 2015).

It is known that non-coding regions of the genome contain regions that perform a wide range of regulatory functions: promoter regions, enhancers, negative regulatory elements, nuclear matrix attachment regions, regions that determine the structure of topologically associating domains (TADs), and other features of 3D organization of genome (Mathelier et al., 2015; Meddens et al., 2019; Ibrahim, Mundlos, 2020). The proportion of regions in the human genome potentially involved in the transcriptional regulation is extremely high. According to the ENCODE project, the chromatin regions corresponding to the peaks of transcription factor(TF) binding identified by the ChIP-seq occupy ~8.1 % of the total genomic DNA (ENCODE Project Consortium, 2012), which is significantly higher than the proportion of coding regions of the human genome (~1.2 %). Considering that not all known TFs and not all cell lines were studied in the ENCODE project, an obviously larger fraction of genomic DNA is involved in the interaction with TFs. The total length of human genome regions with enhancer-associated chromatin features also significantly exceeds the total size of the coding regions: for example, in only one cell type studied (H1-ES), enhancer regions occupy ~3.2 % (Roadmap Epigenomics Consortium et al., 2015).

Studies aimed at identifying the mechanisms of the influence of pathogenic GVs on the predisposition to diseases are carried out very actively, which is reflected in a number of review publications (Mathelier et al., 2015; Merkulov et al., 2018; Smith et al., 2018; Wang et al., 2019; Vohra et al., 2020). The most discussed effect of pathogenic GVs is a change in the binding activity of TFBSs (Lewinsky et al., 2005; Chen L. et al., 2013; Claussnitzer et al., 2015; Mathelier et al., 2015; Gorbacheva et al., 2018). It has also been shown that polymorphic loci can be associated with alteration of DNA methylation patterns (Howard et al., 2014; Kumar D. et al., 2017; Rahbar et al., 2018; Schmitz et al., 2019) and modifications of histone proteins (Kilpinen et al., 2013; Visser et al., 2015; Zhang et al., 2018; Cong et al., 2019), with structural change in chromatin loops (Visser et al., 2015; Zhang et al., 2018) and, as one of the manifestations of this process, with changes in the TADs structure (Cong et al., 2019; Mei et al., 2019). Examples of such effects will be discussed below (Table 1).

**Table 1.**
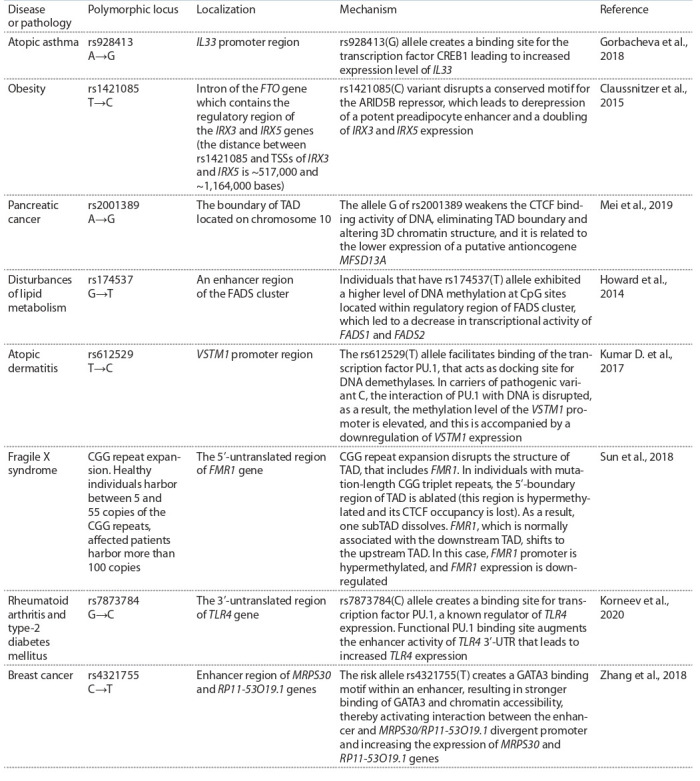
Examples of polymorphic loci associated with pathologies and mechanisms of their action on the gene expression level

**The effects of genetic variants on the functional activity of transcription factor binding sites**

The key role in the transcriptional regulation is played by transcription factors – proteins that can specifically bind to DNA of the regulatory regions of genes and to initiate the transcription complexes formation. The human genome contains more than 1500 genes encoding TFs (Wingender et al., 2013). TF binding sites, as a rule, have a length of 10–25 nucleotides (Levitsky et al., 2014; Kulakovskiy et al., 2018).

Nucleotide substitutions, as well as short insertions/deletions at polymorphic loci, can disrupt TFBSs or create them de novo (see Table 1), and this, in turn, can have both negative and positive effects on the level of gene transcription (Chen L. et al., 2013; Gorbacheva et al., 2018). Such GVs (and the corresponding polymorphic loci) that affect the transcriptional activity of genes are usually called regulatory variants (Kumar S. et al., 2017; Guo, Wang, 2018; Merkulov et al., 2018).

Pathological (that is, associated with a disease) can be both an allelic variant of the DNA sequence containing a disrupted TFBS (Lewinsky et al., 2005; Chen L. et al., 2013; Claussnitzer et al., 2015; Kumar D. et al., 2017; Mei et al., 2019) and an allelic variant, leading to creation of TFBS de novo (Gorbacheva et al., 2018; Zhang et al., 2018; Korneev et al., 2020) (see Table 1).

**Fig. 1.**
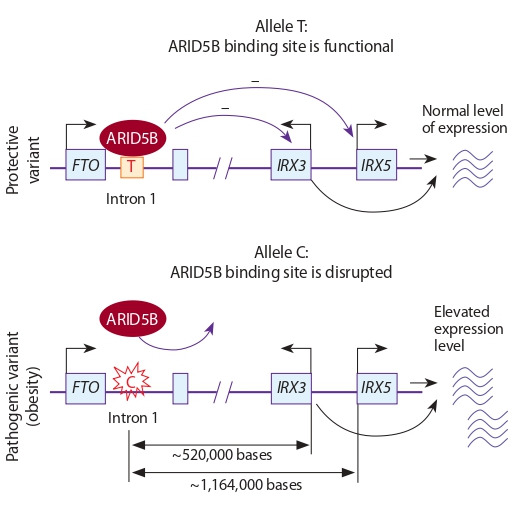
Disruption of the binding site caused by T→C substitution (rs1421085) weakens ARID5B repressor binding to the regulatory region of the IRX3 and IRX5 genes. As a result, the level of expression of IRX3 and IRX5 is increased.

Pathological GVs, affecting the binding activity of TFBSs, can be located not only in promoter regions, but also in regulatory regions located at considerable distance from transcription start sites (TSSs) of genes: enhancers (Lewinsky et al., 2005; Zhang et al., 2018; Meddens et al., 2019), regulatory regions with repressive function (Claussnitzer et al., 2015), and TAD boundary regions (Mei et al., 2019) (see Table 1). For example, the rs1421085 T→C substitution associated with obesity impairs the functioning of the negative regulatory region controlling expression of the IRX3 and IRX5 genes (Claussnitzer et al., 2015). The rs1421085 locus is located in the intron of the FTO gene (Fig. 1) at a considerable distance from the transcription start sites of IRX3 and IRX5 (~520,000 and ~1,164,000 bases). Normally, the DNA region containing allele T interacts with a repressor factor ARID5B, leading to a decrease in transcriptional activity of IRX3 and IRX5 genes. In carriers of the mutant variant of the DNA sequence (allele C), the binding site of the ARID5B repressor factor is disrupted, which causes an excessively high expression of the IRX3 and IRX5 genes and activates adipogenesis (Claussnitzer et al., 2015).

**Fig. 2.**
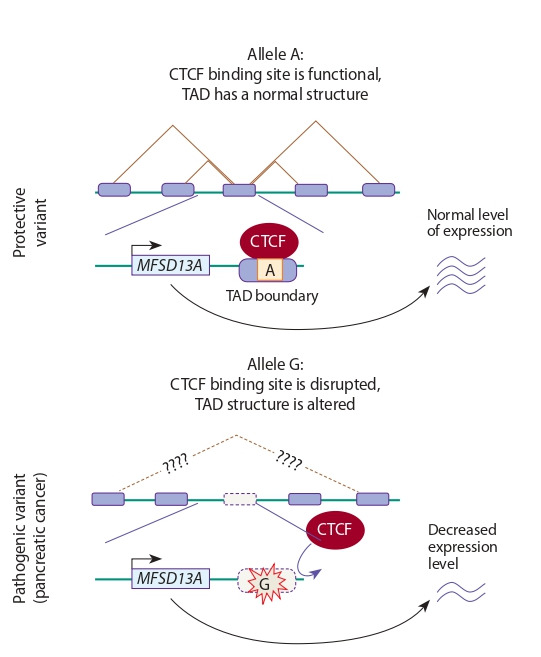
Disruption of the CTCF binding site caused by the nucleotide substitution (rs2001389) eliminates one of the boundary regions that determine the TAD structure. As a result, the tumor suppressor gene MFSD13A expression is downregulated. The contacts between chromatin regions within the TAD are shown with brown lines. Interrogation points in the bottom figure indicate the lack of data on the new structure of TAD.

Occasionally a nucleotide substitution at a polymorphic locus disrupts the TFBS and this, in turn, affects the functional activity of the TAD (see Table 1). This effect was found in the case of A→G (rs2001389), associated with the risk of pancreatic cancer (Fig. 2). The rs2001389 locus is located in the region that determines the structure of chromatin loops within the TAD. This TAD contains 91 genes and is formed by spatially adjacent chromatin regions (Mei et al., 2019). The DNA region containing the risk allele G is characterized by a reduced ability to interact with CTCF, which in this case acts as a structural protein of chromatin. Normally, CTCF binding ensures the functioning of one of the regions that determines the structure of chromatin loops within the considered TAD. The pathogenic allele G alters the activity of CTCF binding motif within TAD boundary disrupting the stability of corresponding 3D structure of chromatin. As a result, the expression of the genes within this TAD is impaired. In this case, the greatest decrease in MFSD13A expression is observed.

**The effects of genetic variability on DNA methylation and gene transcriptional activity**

DNA methylation doesn’t change the nucleotide sequence and is the addition of a methyl group to the fifth carbon atom of cytosine (Angeloni, Bogdanovic, 2019). An increase in the level of DNA methylation, as a rule, leads to a long-term inactivation of the expression of genes lying in the methylated region, since, according to the generally accepted concept, methylation of a DNA region facilitates recruiting protein complexes, containing histone deacetylase (HDAC) (Jones et al., 1998; Nan et al., 1998). DNA methylation can also decrease the ability of some TFs to interact with DNA: it is known that CTCF factors and factors from the ETS family have such sensitivity to methylation (Wang et al., 2019). In contrast, another transcription factor, ZFP57, binds only to methylated DNA (Quenneville et al., 2011). Thus, cytosine methylation can activate different mechanisms of gene transcription regulation, and not always an increase in the methylation level of the regulatory DNA region is associated with a decrease in the expression of the corresponding gene (Izzi et al., 2016; Wang et al., 2019).

Genetic variability affects significantly the methylation of DNA regions that have regulatory potential. Thus, a genomewide analysis of the methylation patterns of DNA collected from 24 subjects from Norfolk Island genetic isolate (Benton et al., 2019), identified 12,761 regions containing at least two CpG dinucleotides and having an allele-specific methylation level. In most cases (98 %), regions with allele-specific methylation level are co-localized with single nucleotide variants presented in dbSNP (Benton et al., 2019).

This study (Benton et al., 2019) also analyzed the location of allele-specific methylation regions relative to the set of polymorphic loci associated with human diseases extracted from the GWAS catalog database. It turned out that polymorphic loci associated with diseases overlap with regions of allele-specific methylation twice more often than it would be expected by chance. This means that the change in methylation levels due to genetic variability is one of the factors that increase the risk of disease.

As an example, consider the rs174537 (G→T) polymorphic locus located in the enhancer of the FADS1 and FADS2 genes encoding fatty acid desaturases 1 and 2. The T variant of the rs174537 locus is associated with an increased risk of pathological disturbances of lipid metabolism (see Table 1). It was shown that individuals that have rs174537(T) allele had a higher methylation level of the regulatory region of the FADS1 and FADS2 genes in human liver (Howard et al., 2014), which led to the suppression of the transcriptional activity of FADS1 and FADS2

**Fig. 3.**
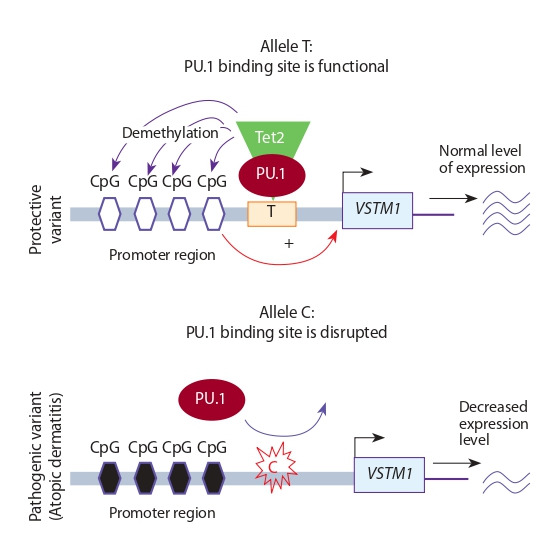
Disruption of the PU.1 binding site caused by the T→C (rs612529) nucleotide substitution reduces the activity of demethylases (for example, Tet2) that maintain the VSTM1 promoter region in an active state, and therefore VSTM1 expression is downregulated.

Occasionally, in one of the allelic variants, DNA demethylation occurs, initiated by TF binding to DNA (see Table 1). For example, such a mechanism was revealed for rs612529 T→C. This locus is located in the promoter region of the VSTM1 (Fig. 3). The low expression of VSTM1 in monocytes provokes the development of atopic dermatitis. In this cell type, the promoter region containing the protective variant T interacts with the transcription factor PU.1 more actively than the other one containing variant C. PU.1 initiates DNA demethylation by recruiting DNA demethylases (for example, Tet2). As a result, carriers of the T allele have completely demethylated VSTM1 promoter, and VSTM1 expression is activated. In carriers of pathogenic variant C, the interaction of PU.1 with DNA is disrupted, as a result, methylation level of the VSTM1 promoter is elevated, and this is accompanied by a decrease in VSTM1 expression (Kumar D. et al., 2017).

**The effects of the genetic variability on the chromatin states and chromatin spatial organization**

Pathogenic GVs may impaire the chromatin state (Kilpinen et al., 2013). There are cases when the presence of a pathogenic GV was accompanied by a change in the patterns of histone modification and the appearance (or disappearance) of DNase I hypersensitive sites (McVicker et al., 2013; Visser et al., 2015; Zhang et al., 2018; Cong et al., 2019). In these cases, allele-specific contacts between promoters and enhancers were identified, the number of which correlated with the activity of the enhancer regions.

**Fig. 4.**
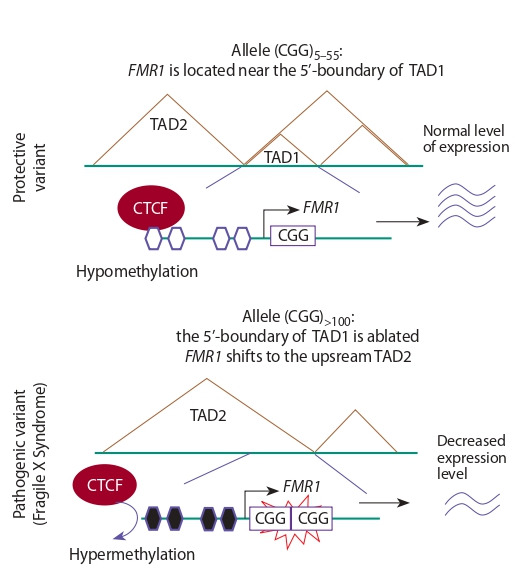
With an increase in the number of CGG triplet repeats in the 5’-untranslated region of the FMR1 gene, the DNA region corresponding to the TAD1 boundary region is hypermethylated. This leads to impaired binding of CTCF factors and disrupts a barrier function of the boundary region. The brown lines show the contacts between chromatin loops within TADs.

There are also known cases when structural variations of the genome (insertions, deletions, duplications, inversions, translocations longer than 50 nucleotides) lead to a change in the spatial organization of chromatin, thereby disrupting the expression of genes associated with pathological processes (Sun et al., 2018; Ibrahim, Mundlos, 2020). For example, the expansion of CGG trinucleotide repeats in the 5′-untranslated region (5′-UTR) of the FMR1 gene, associated with the fragile X syndrome, disrupts the structure of TAD, that includes FMR1 (Fig. 4, see Table 1). Normally, FMR1 is very close to the 5′-boundary region of TAD (in Fig. 4, this is TAD1). The DNA region corresponding to this 5′-boundary is hypomethylated and is occupied by CTCF. In individuals with mutation-length CGG triplet repeats (more than 100), this boundary is ablated (this region is hypermethylated and its CTCF occupancy is lost). As a result, TAD1 dissolves and the boundary of the other TAD (in Fig. 4 it is designated as TAD2) shifts to the 3′-region of FMR1. Therefore, FMR1 is within the TAD2, which normally does not contain this gene. In this case, FMR1 promoter is hypermethylated, and FMR1 expression is inactivated (Park et al., 2015; Sun et al., 2018).

To study molecular-genetic mechanisms of the effect of genome variability on the 3D chromatin structure, it is necessary to reconstruct the spatial genome organization. The following basic levels of the 3D genome organization have been identified: (1) regulatory DNA loops that bring together promoters and enhancers; (2) topologically associating domains(TADs), within which DNA regions have more contacts with each other than with neighboring domains; (3) A and B compartments corresponding to transcriptionally active and condensed chromatin; and finally (4) chromosome territories (Fishman et al., 2018; Hansen et al., 2018). Disruption of 3D contacts between promoters and enhancers within the TAD, caused, for example, by chromosomal rearrangements, can significantly affect the transcriptional activity of a gene, increasing risk of diseases (Lupiáñez et al., 2015).

The Institute of Cytology and Genetics SB RAS has developed an experimental computer approach for prediction physical contacts between promoters and enhancers within the 3D chromatin structure (Fishman et al., 2018; Belokopytova et al., 2020; Belokopytova, Fishman, 2021). The approach is based on the following information: (1) cell type; (2) cell-specific localization of enhancers in the linear genome (from the ENCODE database); (3) transcriptional activity of promoters (from RNA-seq experiments); (4) boundaries of chromatin loop extrusion (based on ChIP-seq mapping of CTCF occupancy in a definite cell type); (5) orientation of CTCF binding motifs (based on motif prediction pipeline); (6)Aor B chromatin compartment (according to Hi-C experiments). Analysis of these data using the original 3DPredictor program (Belokopytova et al., 2020), developed on the basis of machine learning algorithms, allows to predict the frequencies of physical contacts between promoters and enhancers in the 3D genome structure with an accuracy that exceeds the accuracy of other known prediction methods.

The 3DPredictor was used to analyze the 3D genome structure in homozygous DelB/DelB mice that have a deletion of the 1.5 Mb genomic region containing Epha4. This deletion is accompanied by the appearance of additional contacts between Pax3 gene and Epha4 enhancer region, altering Pax3 expression and leading to brachydactyly. Mice with the DelB/ DelB genotype are a genetic model of human pathology accompanied by limb malformations (Lupiáñez et al., 2015). Testing 3DPredictor on this model has demonstrated the high efficiency of the program: in homozygous DelB/DelB mice, ectopic contacts between the Pax3 gene and Epha4 enhancers cluster were predicted (Belokopytova et al., 2020), and these predictions were in good agreement with the experimental data. 

**Genetic variability: combined analysis of heterogeneous big biological and genetic data**

As noted above, many polymorphic loci associated with diseases are located at a considerable distance from the coding regions of genes (ENCODE Project Consortium, 2012; Maurano et al., 2012). Additional studies are needed to identify the molecular-genetic mechanisms of the influence of such GVs on the predisposition to diseases. The purpose of such studies is to clarify the regulatory role of GVs. A typical example is the work (Zhang et al., 2018), which made it possible to find a functionally active regulatory variant rs4321755 associated with the risk of breast cancer. The rs4321755 locus is located in a distant enhancer that regulates the expression of the MRPS30 and RP11-53O19.1 genes (see Table 1). It turned out that in the presence of the pathogenic variant rs4321755(T), a new GATA3 binding site is created. The transcription factor GATA3 increases the functional activity of the enhancer, this leads to the formation of more contacts between the enhancer and the divergent promoter of the MRPS30 and RP11-53O19.1 genes, and increased expression level of these genes. To identify this functionally significant regulatory variant, the authors developed an integrated experimental computer method based on a combined analysis of heterogeneous big biological and genetic data, including: (1) data on allele-specific expression obtained from RNA-seq in combination with data on haplotypes; (2) expression quantitative trait loci (eQTL); (3) genomic distribution of DNAse I hypersensitive sites; (4) localization of ChIP-seq peaks from ENCODE and GEO databases; (5) localization of regulatory motives predicted by computer programs. Similar scenarios for integrated experimental computer research have been implemented in the other studies (Chen C.-Y. et al., 2014; Claussnitzer et al., 2015; Zhao et al., 2019; Li et al., 2020).

This kind of research became possible due to (1) the development of modern high-throughput experimental approaches that allow producing data of different types on a genome-wide scale (parallel high-throughput sequencing, ChIP-seq, 3C, Hi-C, ChIA-PET techniques, DNAse I footprinting, bisulfite sequencing, etc.); (2) development of public information resources accumulating such experimental data. Table 2 provides a brief description of information resources containing genomic data obtained on the basis of omics technologies and used to study the mechanisms by which GVs alter the level of transcription. These resources present (1) the human genome annotation (GENCODE); (2) genome variability in human populations (HapMap, 1000 Genomes Project, IGSR, dbSNP); (3) GVs associated with diseases (GWAS central, GWAS catalog, ClinVar, HGMD, OMIM, etc.); (4) modifications of the chromatin (ENCODE, NIH Roadmap Epigenomics Mapping Consortium); (5) expression quantitative trait loci (GTEx project, eQTL databases, exSNP, etc.); (6) profiling of transcription factor binding events by ChIP-seq (Cistrome Data Browser, GTRD, ReMap); (7) allele-specific binding of TFs, identified using ChIP-seq data in combination with the data on the genotypes of the studied cells (AlleleDB, AlleleSeq); (8) the effects of genetic variability on TFBSs predicted in silico by computer programs (HaploReg, SNP2TFBS, rSNPBase, rVarBase).

**Table 2.**
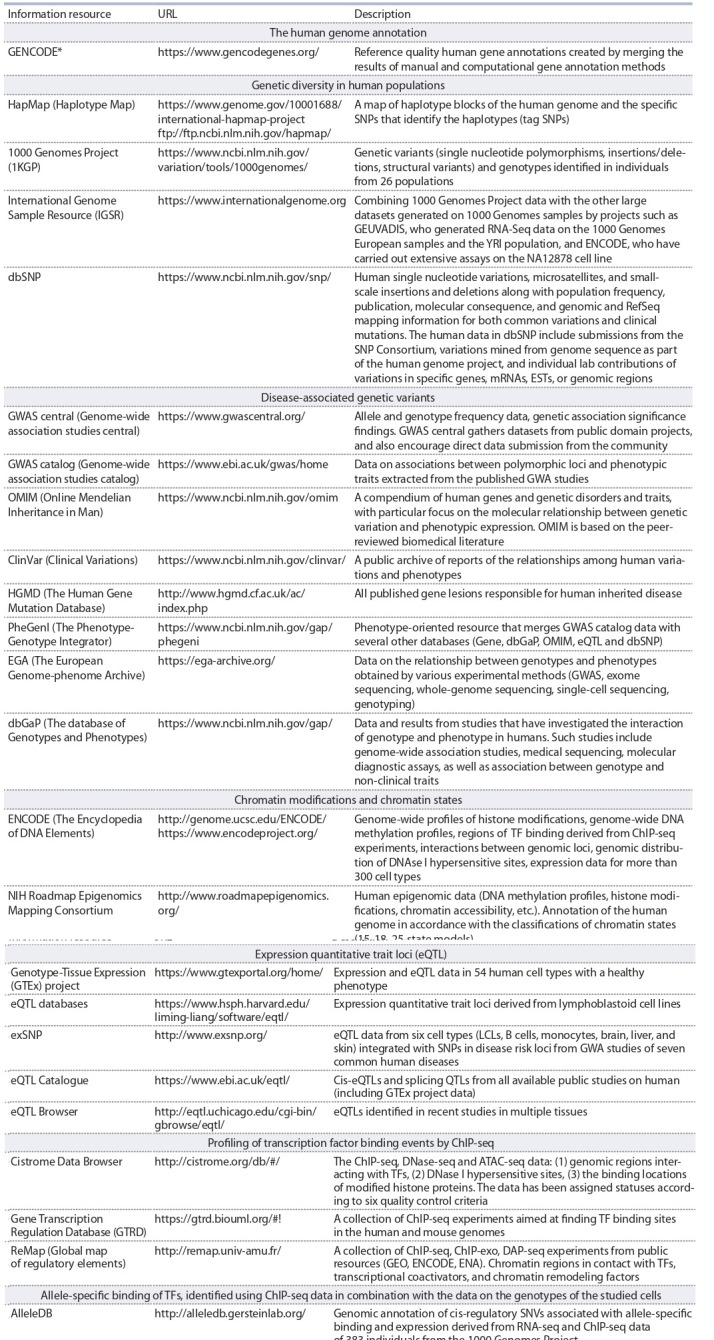
Information resources on genomic data obtained on the basis of the modern high-performance experimental methods * GENCODE reference gene annotations for the human and mouse genomes are also available through the UCSC Genome Browser (https://genome.ucsc.edu/) and the Ensembl genome browser (https://www.ensembl.org/index.html).

**Fig. 5.**
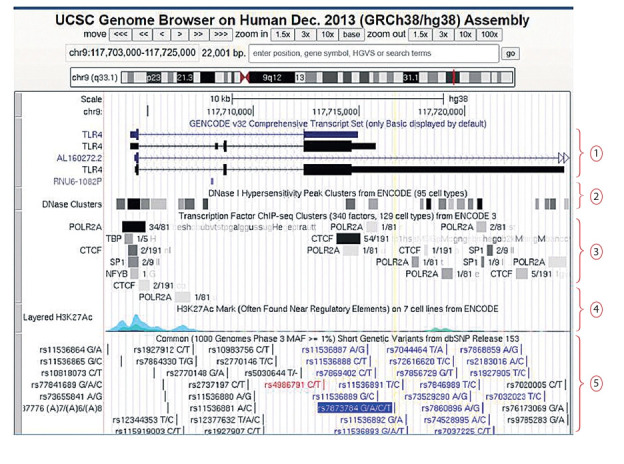
The view of the human genomic region (chromosomal coordinates chr9: 117,703,000–117,725,000) displayed by the Genome Browser of the University of California, Santa Cruz, USA (UCSC Genome Browser, https://genome.ucsc.edu/). (1) transcripts of the TLR4 gene, displayed according to the GENCODE v32 release; (2) DNase I hypersensitivity peak clusters derived from assays in 95 cell types (as a part of the ENCODE project); (3) transcription factor binding derived from a large collection of ChIP-seq experiments performed by the ENCODE project; (4) levels of enrichment of the H3K27Ac histone mark across the genome as determined by a ChIP-seq assay on 7 cell lines from ENCODE (H3K27Ac is the acetylation of lysine 27 of the H3 histone protein, and it is often found near regulatory elements); (5) short genetic variants from dbSNP release 153. The yellow vertical line marks the position the SNP rs7873784 located in the 3’-UTR of TLR4 gene and associated with development of rheumatoid arthritis and type 2 diabetes (see Table 1). According to (Korneev et al., 2020), the G→C substitution at the rs7873784 locus creates PU.1 binding site, that increases the activity of the enhancer located in the 3’-UTR of the TLR4 gene.

A separate category of information resources includes: (1) the genome browser of the University of California, Santa Cruz, USA (UCSC Genome Browser, https://genome.ucsc.edu/) and (2) the genome browser of the Ensembl database which is a joint research project of the European Bioinformatics Institute and the Wellcome Trust Sanger Institute (Ensembl Genome Browser, https://www.ensembl.org/index.html). These genome browsers integrate data on genome sequences and its features obtained by different research groups using a wide range of experimental methods (Lee et al., 2020; Yates et al., 2020). The websites of these browsers provide access to the primary DNA sequences and genome annotations for many organisms (including vertebrates and several other model species). Browser’s graphical interfaces allow to obtain scalable maps of genomic regions and to visualize interactively a large number of annotations and features (for example, positions of transcripts, positions of GVs, chromatin regions interacting with TFs detected by ChIP-seq experiments, data on genome-wide mapping of DNase I hypersensitive sites, etc.) (Fig. 5).

The websites of the UCSC Genome Browser and Ensembl Genome Browser provide access to software tools for extraction data as text files: UCSC table browser (https://genome.ucsc.edu/cgi-bin/hgTables) and BioMart data mining tool (https://www.ensembl.org/info/data/biomart/index.html).

**Information resources on allele-specific binding of transcription factors and on the effects of genetic variants on TFBSs predicted in silico**

As noted above, the influence of pathogenic GVs on gene expression is often mediated through a change in the functional activity of TFBSs. In this regard, information resources that include whole genome data on allele-specific binding of TFs, identified based on the ChIP-seq method, can be extremely useful. A range of approaches have been developed to identify allele-specific binding of TFs (Rozowsky et al., 2011; Reddy et al., 2012; Waszak et al., 2014; Younesy et al., 2014). These approaches are based on the analysis of the ChIP-seq data in combination with the sequencing data, which allow to find heterozygous loci within a single genome and to phase genotypes of the studied cells. Thus, for each type of cells examined, its own set of genomic loci interacting with a specific transcription factor in an allele-specific manner can be identified. For example, in (Cavalli et al., 2016a), the ChIP-seq data for 55 TFs in the HepG2 cells and 57 TFs in the HeLa-S3 cells were analyzed. In HepG2 cells, 3001 genomic loci with allele-specific signals were found, and 712 loci were found in HeLa-S3 cells. The authors note the pronounced tissue-specific nature of allele-specific TF binding: of the entire set of identified loci, only 34 were found in both cell lines (Cavalli et al., 2016a).

The data on allele-specific binding of TFs are collected in the following information resources: AlleleDB http://alleledb.gersteinlab.org/ (Chen J. et al., 2016), AlleleSeq (http://alleleseq.gersteinlab.org/) (Rozowsky et al., 2011) (see Table 2), as well as in the supplemental files to publications (Cavalli et al., 2016a, b, 2019; Shi et al., 2016).

Studies aimed at identifying allele-specific TF binding made it possible to estimate the number of genetic variants that affect the binding of a particular transcription factor to DNA in a particular cell type. The average number of such events registered for a single transcription factor can range from 19 to 37 for cells with a normal karyotype (GM12878, H1-hESC) and from 12 to 55 for cancer cell lines (SK-N-SH, K562) (Cavalli et al., 2016a, b).

When generating hypotheses on the mechanisms that mediate the effect of GVs on disease risk, one can also use the data on the effects of genetic variants on the functional activity of TFBSs predicted in silico. Such information is accumulated in specialized databases: HaploReg (https://pubs.broadinstitute.org/mammals/haploreg/haploreg.php) (Ward, Kellis, 2012), SNP2TFBS (http://ccg.vital-it.ch/snp2tfbs/) (Kumar S. et al., 2017), rSNPBase (http://rsnp3.psych.ac.cn/index.do) (Guo, Wang, 2018), rVarBase (http://rv.psych.ac.cn) (see Table 2).

## Conclusion

A significant proportion of pathogenic genetic variants associated with diseases are located in non-coding regions of the human genome. Such genetic variants can with a high degree of probability disrupt functional activity of regulatory regions that control the transcriptional activity of genes. The examples of the mechanisms of influence of pathogenic genetic variants on gene expression considered in this review confirm this possibility. The studies that have made it possible to identify these mechanisms are complex and are based on the analysis of big heterogeneous genetic data. The online omics data resources provide ample opportunities for such research. Further development of experimental techniques and bioinformatics methods for analyzing the data obtained with the help of this techniques, as well as an increase in the set of investigated cell types, will significantly expand these possibilities.

## Conflict of interest

The authors declare no conflict of interest.
